# Clonal Evolution and Changes in Two AML Patients Detected with A Novel Single-Cell DNA Sequencing Platform

**DOI:** 10.1038/s41598-019-47297-z

**Published:** 2019-07-31

**Authors:** Liwen Xu, Robert Durruthy-Durruthy, Dennis J. Eastburn, Maurizio Pellegrino, Omid Shah, Everett Meyer, James Zehnder

**Affiliations:** 1 Department of Pathology, Stanford University, Stanford, CA 94305 USA; 2 Mission Bio, Inc., South San Francisco, CA 94080 USA; 3 Division of Blood and Marrow Transplantation. Department of Medicine, Stanford University, Stanford, CA 94305 USA

**Keywords:** Cancer genomics, Cancer genomics

## Abstract

Next-generation sequencing (NGS) is used to detect gene variants in genetically complex cell populations of cancer patient samples. Traditional bulk analysis can only provide average variant allele frequencies of the targeted genes across all sampled cells. It fails to resolve mutational co-occurrences and may miss rare cancer cells. Genome analysis at the single cell level offers the opportunity to more fully resolve clonal architecture. Peripheral blood mononuclear cells were sampled from acute myeloid leukemia patients longitudinally and single-cell DNA sequencing libraries were generated with a novel droplet-based microfluidics approach. Molecular profiling of single nucleotide variants across thousands of cells revealed genetic chimerism in patients after bone marrow transplantation (BMT). Importantly, hierarchical clustering analysis of single nucleotide variants (SNVs) uncovered a distinct oncogenic clone of cells carrying mutated tumor-suppressor and/or oncogene(s). This novel single-cell DNA sequencing approach enabled precise monitoring of engraftment and revealed clonal evolution of oncogenic cells during the progression and treatment of the disease.

## Introduction

Acute myeloid leukemia (AML) is a clinically and biologically heterogeneous hematologic malignancy characterized by clonal expansion of undifferentiated myeloid precursors, which results in impaired hematopoiesis and bone marrow failure. Two or more driver mutations are frequently observed in one or multiple genes in AML. The most commonly mutated genes in AML are the tumor suppressor and DNA repair gene *TP53* and genes involved in signal transduction, DNA methylation, regulation of RNA transcription and splicing, and chromatin modification refs^1–7^. Furthermore, it has been reported that the spectrum of driver gene mutations is linked to prognostic outcomes refs^4–6,8,9^. Currently, bulk next-generation DNA sequencing (NGS) is used to detect gene variants in genetically and phenotypically complex cell populations of AML patient samples. This bulk NGS analysis can only provide average variant allele frequencies (VAF) of the targeted loci across all cells in the whole clinical samples. It fails to resolve co-occurrence patterns of gene mutations in the same cells, is unsuccessful in resolving zygosity states and may miss rare cancer cells, which are often implicated in disease emergence and relapse. Therefore, high-throughput genomic analysis strategies at the single cell level are needed to study genetically heterogeneous cells in AML clinical samples.

Recently, single-cell sequencing has emerged as a promising approach to study cancer and to further understand the disease refs^10–14^. Most available single-cell NGS strategies aim to amplify the entire genome and/or only profile a low number of cells per sample with laborious workflows. This leads to either considerable levels of technical artifacts (e.g., high allele dropout events and non-uniform coverage) or insufficient cell numbers that may not be representative of biological samples.

In this study, we used a novel two-step droplet microfluidics approach that enables to profile genomic alterations across thousands of cells in targeted and automated fashion ref.^15^. Using the Tapestri Platform we analyzed peripheral blood mononuclear cells (PBMCs) from two AML patients longitudinally at three distinct time-points: before bone marrow transplant (pre-BMT), after bone marrow transplant (post-BMT) and at AML relapse (relapsed-AML). The single-cell DNA-sequencing (DNA-seq) data allowed us to directly assess donor/host chimerism using the individuals’ unique genotype signatures as genetic proxies. We successfully identified all bulk DNA-seq verified mutations in the single-cell DNA-seq data and showed that number and frequency of variants corroborated bulk NGS data. Importantly, we identified a unique clone of oncogenic cells that can’t be detected with conventional bulk sequencing. Comparison of clone number and size across all three time-points in each patient suggested that AML relapse after bone marrow transplantation (BMT) may result from the aggressive and exclusive expansion of the oncogenic cells which carry tumor-suppressor gene and/or oncogene mutation(s) and are associated with loss of donor chimerism.

## Results

### A novel droplet microfluidics approach to detect gene mutations at single cell level

The single-cell platform we used in this study facilitates a novel two-step droplet microfluidics approach to detect genomic DNA alterations (single nucleotide variants (SNVs) and short indels) across thousands of cells at single cell level in targeted, scalable and automated fashion. First, thousands of cells were encapsulated and lysed in picoliter-sized droplets and subsequently protease-treated to liberate DNA from histones and other DNA-binding proteins. Secondly, individual cell lysates were uniquely barcoded and a total of 40 amplicons spanning 19 AML-specific genes plus 10 control amplicons were simultaneously PCR-amplified inside each droplet (Supplementary Figure 1). This barcoding strategy preserved each cell’s mutational profile and allowed all cells to be pooled and processed together. Lastly, amplified products were prepared with conventional sequencing library chemistry, single-cell sequencing libraries were sequenced on a MiSeq instrument and the data was processed and analyzed with Mission Bio’s cloud-based analysis software platforms (Supplementary Figure 1).

In this study, a total of 2,045 to 8,619 single cells per sample were processed, sequenced and analyzed. After removing low quality cells, we focused our downstream analyses on ~1,500–~4,700 cells per sample. Allele dropout rates ranged from 5.3% to 12.0% across all six samples measured with the help of control amplicons (Supplementary Figure 2).

### Single-cell DNA sequencing revealed bone marrow (BM) engraftment efficiencies and changes of BM chimerism over time during progression of AML

Hierarchical clustering of the single-cell data using both well-annotated common SNVs and disease-related somatic variants revealed genetic chimerism in both patients after BMT and allowed us to definitively differentiate patient-derived cells from donor-derived cells in both post-BMT and relapsed-AML samples. As a result, we were able to assess bone marrow (BM) engraftment efficiencies and monitor the changes of genetic mosaicism over time, which differed substantially between both patients.

**Figure 1:**
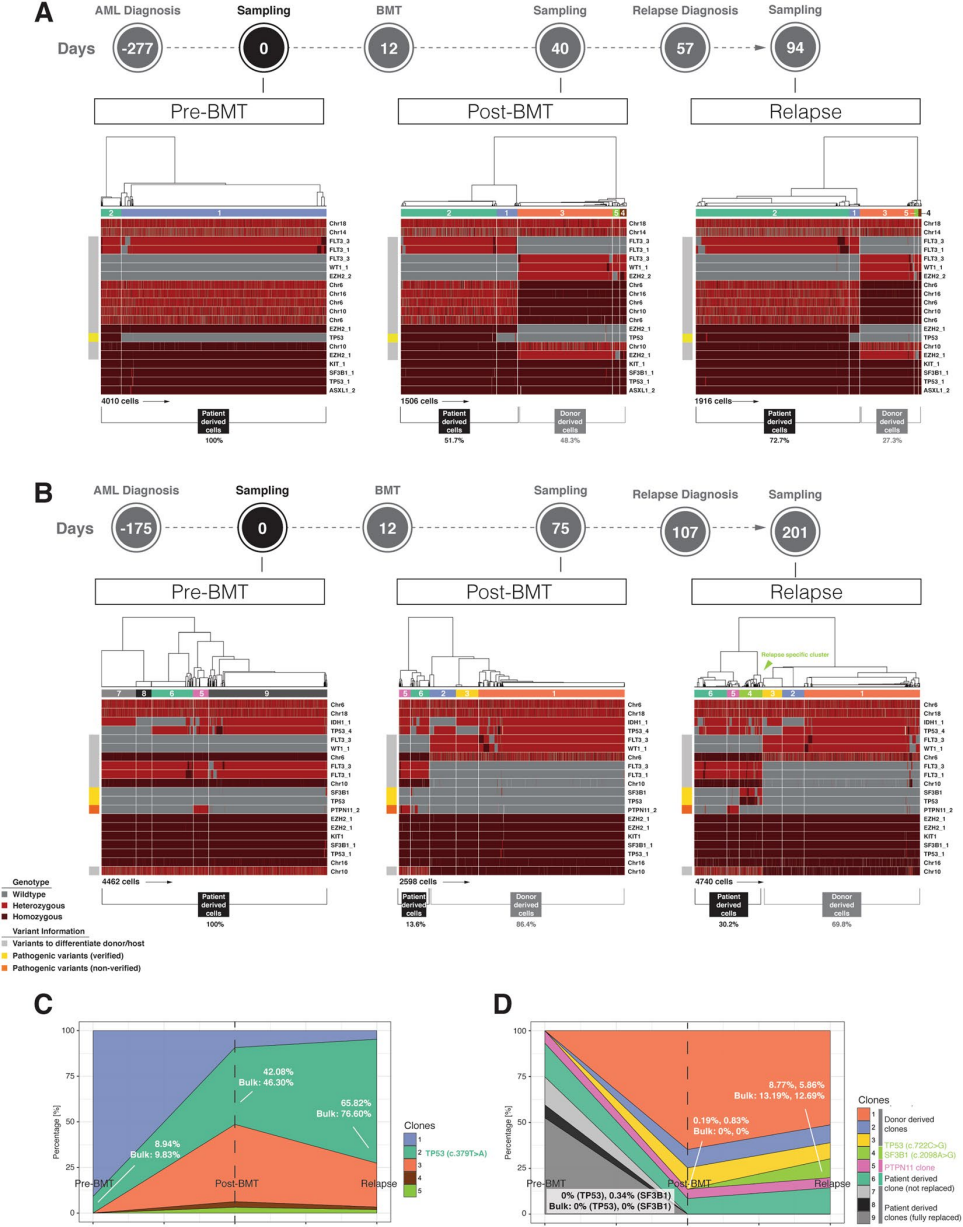
Hierarchical clustering of SNVs and clonal evolution in PBMCs of two AML patients. (**A**) Hierarchical clustering of SNVs in PBMCs of Patient 1. Wild-type genotypes are in grey, heterozygous SNVs in red, and homozygous SNVs in dark red. Different variants are colored in different colors in the boxes on the left side of each heatmap: verified pathogenic variants are colored in yellow; non-verified pathogenic variant is in orange; variants used to differentiate donor from patient cells are in grey. Different clones of cells based on SNV profiles are shown in different colors in the bars above each heatmap. (**B**) Hierarchical clustering of SNVs in PBMCs of Patient 2. The colors are the same as Panel A. (**C**) Clonal evolution in PBMCs of Patient 1. The colors of different clones are the same as the ones in the bars above the heatmaps in Panel A. (**D**) Clonal evolution in PBMCs of Patient 2. The colors of different clones are the same as the ones in the bars above the heatmaps in Panel B.

In Patient 1, we found a total of 20 high-quality SNVs including 19 common variants and 1 pathogenic variant (Supplementary Table 3A). Before transplantation, PBMCs contained two main clones of cells: a small clone (clone #2, green) of cells carrying a missense *TP53* mutation (c.379 T > A); representing putatively the disease-related clone and a large clone (clone #1, blue) of cells containing wild-type (WT) *TP53* (Left panel in Fig. 1A). Using genotype information from 13 common SNVs that differentiated between donor and host allowed us to calculate the bone marrow engraftment efficiency at post-BMT and relapsed-AML stages. At post-BMT, bone marrow engraftment efficiency was ~ 50% (48.3% of cells derived from donor and 51.7% of cells derived from patient). However, at relapsed-AML, donor-derived cells were significantly decreased to 27.3% compared to 48.3% at post-BMT, indicating loss of donor chimerism (Middle and right panels in Fig. 1A).

Results from chimerism analysis using standard clinical short tandem repeat (STR) tests were consistent with our single-cell approach that utilizes differential germline-associated genomic signature information across different specimens (Table 1).

**Table 1:** Changes of chimerism detected in PBMCs of Patient 1 with clinical STR analysis and single-cell DNA sequencing. Chimerism detected from exactly the same sample is bolded.

Clinical Status	Post-BMT	Relapsed-AML	Relapsed-AML
Blood Collection Date	**10/2/15**	11/25/15 (*single-cell)*	11/30/15 (*bulk*)
STR (donor/recipient)	**44%/56%**	N/A	6%/94%
Single-cell Sequencing (donor/recipient)	**48.3%/51.7%**	27.3%/72.3%	N/A

For Patient 2, we applied a similar strategy to measure the overall engraftment efficiency after BMT by leveraging the genomic signature of 7 discriminative common SNVs (Supplementary Table 3B).

After BMT, the patient-derived cells comprised 13.6% of the whole cell population, indicating about 86% BM engraftment. Similar to Patient 1, the donor-derived cell population declined over time from 86.4% at post-BMT to 69.8% at relapsed-AML, indicating loss of donor chimerism (Fig. 1B). Clinical STR test results again agreed with the single-cell data (Table 2).

**Table 2:** Changes of chimerism detected in PBMCs of Patient 2 with clinical STR analysis and single-cell DNA sequencing. Chimerism detected from exactly the same sample is bolded.

Clinical Status	Post-BMT	Post-BMT	Relapsed-AML
Blood Collection Date	2/3/12 (*bulk*)	2/10/12 (*single-cell)*	**6/15/12**
STR (donor/recipient)	94%/6%	N/A	**79%/21%**
Single-cell Sequencing (donor/recipient)	N/A	86.4%/13.6%	**69.8%/30.2%**

It has been reported that mixed chimerism, especially low donor chimerism levels are associated with AML relapse and mortality refs^16,17^. Similarly, we found gradual loss of donor chimerism from post BMT to AML relapse in both patients (Fig. 1, Tables 1 and 2). Moreover, Patient 1 had remarkably lower donor chimerism and took significantly shorter time to relapse after BMT compared to Patient 2 (Tables 1 and 2 and Supplementary Table 1), which also agreed with the previous reports refs^16,17^.

### Single-cell DNA sequencing unveiled exclusive and rapid expansion of the oncogenic clone of cells throughout AML progression

Comparative hierarchical cluster analysis of all 20 SNVs across all three time-points in each patient uncovered a distinct clone of cells carrying mutated tumor-suppressor and/or oncogene(s) that exclusively expanded over time and were remarkably enlarged in the relapsed-AML samples, indicating the presence of cancer cells (Fig. 1).

In Patient 1, single-cell SNV profiling revealed a clone of oncogenic cells carrying *TP53* mutation which was detected in all three time-points and which markedly increased in size over time (Fig. 1A). Clonal evolution analysis showed that out of all the clones identified in this patient, exclusively this mutant *TP53* clone expanded significantly over time, from about 9% at pre-BMT, to about 42% at post-BMT and to almost 70% of the cell population when AML relapsed (Fig. 1C). As shown in Table 3, VAFs for *TP53* mutation calculated from single-cell data and bulk data were comparable at all time-points for this patient.

**Table 3:** Comparison of VAFs in PBMCs of Patient 1 between the bulk sequencing data and the aggregated single-cell sequencing data. The genes detected in both the bulk and the single-cell sequencing are bolded.

Mutation	Function	Bulk	Single-cell	Bulk	Single-cell	Bulk	Single-cell
Clinical Status		Pre-BMT	Pre-BMT	Post-BMT	Post-BMT	Relapsed-AML	Relapsed-AML
TP53 (c.379 T > A)	**Missense**	**9.83%**	**8.94%**	**46.30%**	**42.08%**	**76.60%**	**65.82%**
DNMT3A (c.2099 C > T)	Missense	6.20%	N/A	23.30%	N/A	41.00%	N/A

While conventional clinical tests, such as blast counts in bone marrow or blood failed to detect progression of the disease after BMT compared to pre-BMT (Supplementary Table 4A), single-cell DNA-seq data successfully unveiled disease progression from pre-BMT to AML relapse as indicated by the expansion of the mutant *TP53* oncogenic clone of cells (Fig. 1C).

**Figure 2:**
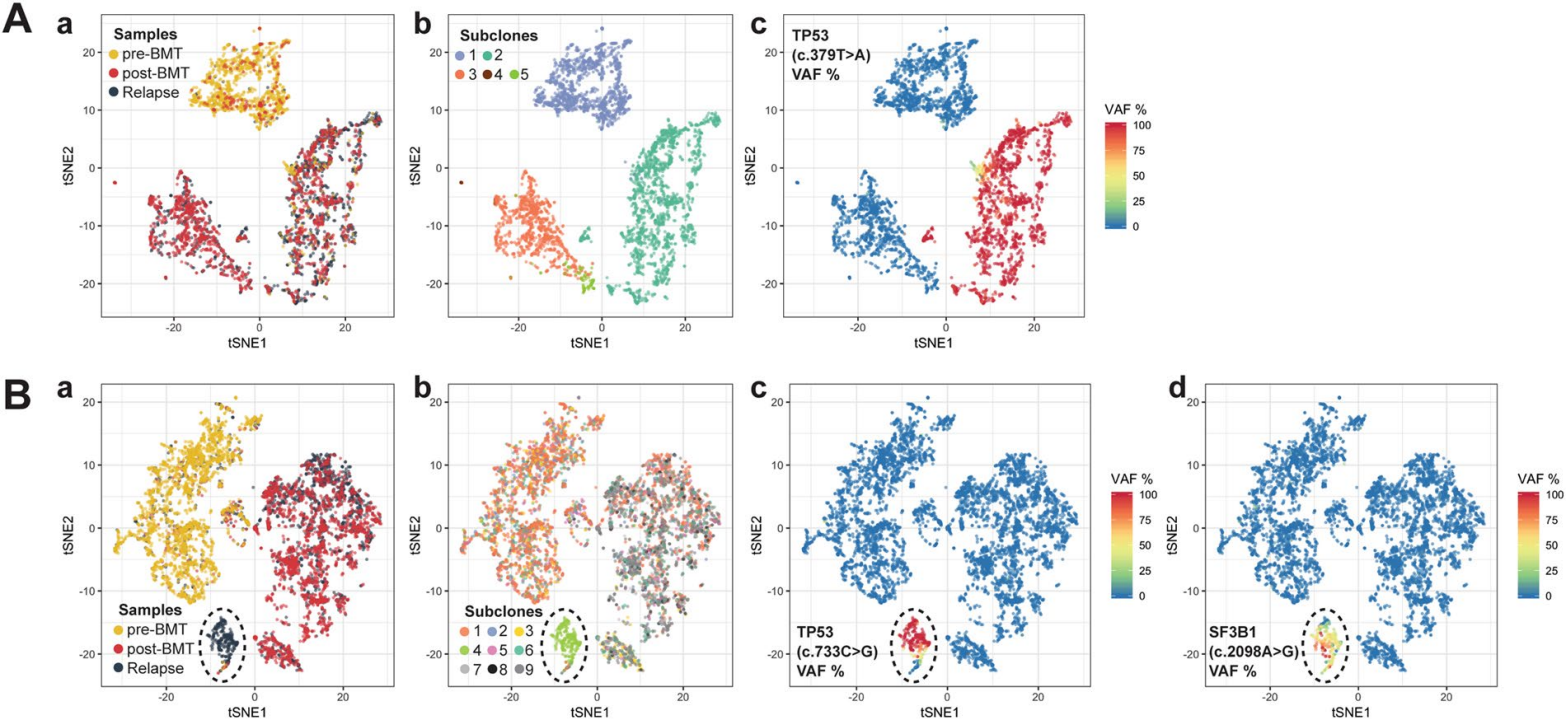
Dimension reduction (t-SNE) of combined PMBCs across three time-points. (**A**) t-SNE of combined PBMCs across three time-points from Patient 1. (a) Overlay of cells from three time-points. Three main clusters are detectable, two of which are primarily comprised of cells from post-BMT and Relapse (red, gray) and one largely represents pre-BMT derived cells (yellow). (b) Overlay of different clones of cells. The colors of the clones match those shown in the bars above the heatmaps in Fig. 1A. (c) TP53 allele frequency status in the cells. Wild-type TP53 is shown in blue, heterozygous TP53 SNV in yellow, and homozygous TP53 SNV in red. (**B**) t-SNE of combined PBMCs across three time-points from Patient 2. The unique oncogenic clone of cells carrying *SF3B1* and *TP53* mutations and being detected in relapsed-AML sample is circled. (a) Overlay of cells from three time-points. Three main clusters are detectable: one group primarily comprised of cells from pre-BMT (yellow), one group largely made up of post-BMT cells (red) with a few Relapse cells (gray) and one small group of largely Relapse derived cells (gray and circled). (b) Overlay of different clones of cells. The colors of the clones match those shown in the bars above the heatmaps in Fig. 1B. (c) TP53 allele frequency status in the cells. The color code is identical to (**A**)(c). (d) SF3B1 allele frequency status in the cells. The color code is identical to (**A**)(c).

To further investigate the relationship between all cells across all three time-points in this patient, we employed t-distributed stochastic neighbor embedding (t-SNE) analysis, an approach that reduces the dimensionality of the high-dimensional data and enables the data to be visualized in two-dimensional space. t-SNE analysis corroborated hierarchical clustering and identified three major groups of cells; one primarily comprised of cells derived from pre-BMT and the other two groups largely associated with cells sampled from both post-BMT and relapsed-AML (Fig. 2A(a)). All three groups of cells were genetically distinct (Fig. 2A(b)). Cells in group 1 represented mainly the patient-derived WT *TP53* clone. Group 2 associated cells represented primarily the donor-derived WT *TP53* clone, whereas group 3 associated cells represented exclusively the mutated *TP53* clone (Fig. 2A(a),(c)).

In Patient 2, PBMCs sampled at AML relapse included a unique oncogenic clone of cells carrying missense mutations in both the *SF3B1* gene (c.2098 A > G) and the tumor-suppressor gene *TP53* (c.722 C > G) (Fig. 2B). Importantly, this *TP53* mutation was undetectable in the pre-BMT sample and was barely detectable (~0.2% VAF) in the post-BMT sample, but it was greatly increased (~9% VAF) in the relapsed-AML sample (Table 4 and Fig. 1B). These results suggested that this *TP53* mutation was either newly acquired after BMT or it originally existed prior to BMT as the founding clone of the minimum residual disease (MRD) and may be detectable by sampling more cells. Notably, conventional bulk sequencing did not detect this mutation either before or after BMT due to its limited sensitivity. Single-cell VAFs at AML relapse correlated well with bulk-sequencing measurements although they were lower compared to bulk sequencing data (Table 4). This discrepancy between single-cell sequencing and bulk sequencing may be explained by a number of factors including non-identical sample starting material and differently used genotype callers.

**Table 4:** Comparison of VAFs in PBMCs of Patient 2 between the bulk sequencing data and the aggregated single-cell sequencing data. The genes detected in both the bulk and the single-cell sequencing are bolded.

Mutation	Function	Bulk	Single-cell	Bulk	Single-cell	Bulk	Single-cell
Clinical Status		Pre-BMT	Pre-BMT	Post-BMT	Post-BMT	Relapsed-AML	Relapsed-AML
TP53 (c.722 C > G)	**Missense**	**Undetected**	**Undetected**	**Undetected**	**0.19%**	**13.19%**	**8.77%**
SF3B1 (c.2098 A > G)	**Missense**	**Undetected**	**0.34%**	**Undetected**	**0.83%**	**12.69%**	**5.86%**
STAG1 (c.1031 G > A)	Missense	Undetected	N/A	Undetected	N/A	6.16%	N/A

Clonal evolution analysis showed that similar to Patient 1, among all the clones of cells identified in this patient, only the oncogenic clone of cells carrying both *TP53* and *SF3B1* mutations aggressively and exclusively expanded during disease progression from post-BMT to relapsed-AML, suggesting this clone of cells may be the cause of AML relapse in this patient (Fig. 1D).

Although clinical tests were unsuccessful in detecting changes of disease status at post-BMT compared to pre-BMT (Supplementary Table 4B), single-cell DNA-seq uncovered expansion of the oncogenic clone of cells carrying both *TP53* and *SF3B1* mutations from 0% before BMT to ~0.2% after BMT and then expansion to ~6% at AML relapse (Fig. 1D and Supplementary Table 4B), implying that exclusive and remarkable expansion of this oncogenic clone of cells might be the cause of AML relapse in this patient.

We also found a mutation in the *PTPN11* gene at all three time-points of patient 2 (Fig. 1B). This particular gene mutation (c.155 C > T) has been reported as likely pathogenic for Noonan Syndrome ref^18^. However, unlike the oncogenic clone of cells carrying both *SF3B1* and *TP53* mutations, this putative pathogenic clone of cells did not expand much during AML progression (~5% VAF at all time-points), (Fig. 1B, 1D), indicating that this particular clone was not associated with AML relapse.

t-SNE analysis unveiled one small and two large groups of cells (Fig. 2B). It revealed that pre-BMT cells were very different from both post-BMT cells and relapsed-AML cells. Moreover, it showed that the clone of cells carrying both *SF3B1* and *TP53* mutations was distinct from the rest and only identifiable in the relapsed-AML sample (Fig. 2B), confirming hierarchical clustering analysis results and highlighting the uniqueness of this clone of cells.

Taken together, the results from single-cell DNA sequencing suggested that the sole and remarkable expansion of the cells carrying both *TP53* and *SF3B1* mutations might be the cause of AML relapse in this patient.

## Discussion

AML is a cancer frequently driven by chromosomal translocations or somatic mutations, which can demonstrate complex clonal diversity refs^19–21^. It has been demonstrated that progression and relapse of AML can be driven by selection and expansion of resistant clones that evade therapeutics refs^10,11,21–25^. Therefore, studying the clonal architecture of tumors requires sensitive and high-resolution approaches that help contribute to a better understanding of tumor heterogeneity and cancer progression. Profiling the genetic architecture of cancer samples by sequencing them at single cell level across thousands of cells provides a powerful strategy to accurately reveal clonal architecture of tumors.

Here we used a novel droplet-based single-cell platform to profile SNVs and small indels in thousands of PBMCs including before BMT, after BMT, and at AML relapse in two AML patients. In both cases we were able to precisely assess BM engraftment efficiencies and monitor the changes of genetic mosaicism over time by leveraging the unique genotype profile information to discriminate between patient-derived and donor-derived cells. Importantly, we were able to identify a distinct clone of cells carrying mutated tumor suppressor and/or oncogene(s) that exclusively expanded over time and that remarkably enlarged in the relapsed-AML samples of both patients. Meanwhile, all other clones of cells, including a *PTPN11* clone (c.155 C > T) in patient 2, a pathogenic variant that has been reported to be linked to Noonan Syndrome, did not expand over time.

**Figure 3:**
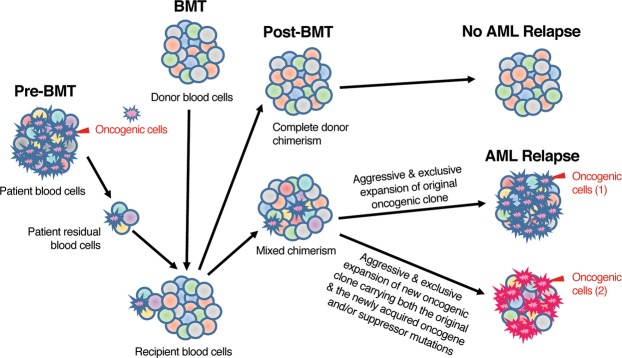
Model of AML relapse in bone marrow transplanted AML patients. In bone marrow transplanted AML patients, AML relapse is caused by the aggressive and exclusive expansion of either (1) the patients’ original oncogenic clone (for MRD) or (2) the new oncogenic clone carrying both the original and the newly acquired tumor suppressor gene and/or oncogene mutations.

We hypothesize that in bone marrow transplanted AML patients, successful BM engraftment with complete donor chimerism will lead to complete remission without AML relapse. On the other hand, incomplete BM engraftment with mixed chimerism will result in gradual loss of donor chimerism and relapse of AML after BMT. In this situation, there are two possible causes of AML relapse: (1) The patients’ original oncogenic clone of cells still exist after BMT and are resistant to therapies. This original oncogenic clone of cells become the founding clone of MRD. They expand exclusively and aggressively which lead to AML relapse. (2) The patients’ original oncogenic clone of cells acquire new gene mutation(s) after BMT. The new oncogenic clone of cells carrying both the original and the newly acquired tumor suppressor gene and/or oncogene mutations expand exclusively and aggressively, thereby leading to AML relapse (Fig. 3).

Intratumor heterogeneity is one of the key hallmarks of cancer. Reconstructing the complex clonal architecture using bulk NGS data has been challenging and depends on prior defined models that include hypotheses of zygosity (often times assumed to be heterozygous) and VAF distributions (often times two or more variants with comparable VAFs assumed to occur in the same subset of cells). On the other hand, the single-cell approach used in this study is independent from such models because each individual cell can be directly measured.

For example, patient 1 in this study exhibited a mutation in *TP53* gene with bulk-measured VAFs expanding from 9.83% before transplantation to 46.3% after transplantation. Assuming the variant is presented heterozygous, the inferred proportion of malignant cells before and after BMT corresponds to ~20% and ~92%, respectively (clonal frequency is twice the variant allele frequency) ref^12^. In contrast, single-cell data clearly demonstrated that only ~10% and 40% of cells carried the mutant allele at pre-BMT and post-BMT, respectively (Supplementary Figure 3D). The discrepancy between both approaches is linked to the zygosity state of the *TP53* mutation. Our single-cell sequencing data showed that at each time-point only the mutated allele was detected in the cells carrying *TP53* mutation, which was indicative for at least one of the three possibilities: (1) presence of true homozygosity, (2) loss of heterozygosity (LOH) with the wild-type allele lost, or (3) presence of true heterozygosity with unbalanced dropout of the wild-type allele. In our study, the distribution of reads across all cells indicated that cluster formation did not depend on read depth, a metric that impacts the ability to robustly call genotypes. In particular, cells in which only the mutant allele of *TP53* was detected did not suffer from lower number of sequencing reads compared to cells that exclusively carried the wild-type allele (Supplementary Figure 3A). Quantitative comparison of sequencing reads between *TP53* mutant and *TP53* wild-type cells showed no significant differences between both distributions in each of the three time-points (Supplementary Figure 3B). In addition, overall variant quality was similarly high when compared to all post-filtering variants (Supplementary Figure 3C). Therefore, allele dropout was unlikely to exclusively impact *TP53* zygosity based on overall variant quality and ~10% ADO rate as measured with control amplicons (Supplementary Figure 2 and 3). Besides, failure to detect one mutant allele in each cell assumes that the putatively true aggregated single-cell VAFs (pseudo-bulk) would be roughly double the measured values, contradicting the bulk measurements. Instead of allelic dropout, the single-cell sequencing data strongly suggested that the wild-type allele was lost during disease progression or the variant truly existed homozygously, as it was previously reported in the context of mutant stabilization refs^26,27^.

This single-cell DNA sequencing platform is a sensitive and robust technology to detect gene mutations at single cell level in AML clinical samples, in excellent agreement with bulk NGS mutation detection. This method also provides an assessment of donor chimerism comparable to existing clinical short tandem repeat-based methods. Importantly, it can provide a precise picture of bone marrow engraftment and mutational profile of tumor cells from one assay simultaneously. Moreover, single-cell data enables the direct measurement of co-occurrence patterns of multiple variants across cells, something that can only be inferred with bulk-sequencing. Particularly in the cases with more than two disease-relevant mutations with varying allele frequencies it becomes challenging to accurately determine the mutational distribution pattern across sub-clones when using traditional bulk sequencing approaches alone. In addition, single-cell technologies reveal cellular zygosity information, which is critical in correctly establishing population frequencies of disease clones and formulating overall disease models.

In addition to identifying the repertoire of somatic variants as with bulk sequencing methods, this single-cell sequencing technology offers direct insight into the clonal architecture and complexity of AML, provides precise monitoring of bone marrow engraftment and reveals clonal evolution of oncogenic cells during the progression and treatment of the disease. It is likely to have utility in disease monitoring and therapeutic strategies.

## Materials and Methods

### Patient characteristics

This study was approved by the Institutional Review Boards of Stanford University. Patient samples were collected with informed and signed consent. All experiments were performed following the guidelines and regulations of the Institutional Review Boards of Stanford University. Information of sample collection timelines, disease progressions and treatment regiments for two patients in this study is shown in Supplementary Table 1. All clinical tests and assays were performed in the Stanford Clinical Molecular Genetic Pathology Laboratory.

### Short tandem repeat (STR) analysis

STR Analysis was conducted in the Stanford Clinical Molecular Genetic Pathology Laboratory. Briefly, genomic DNA was extracted from the white blood cells in peripheral blood samples. Amplifications of extracted DNA were conducted with GenePrint24 kit following the manufacturer’s instruction (Promega Corp, Madison, WI). Detection and analysis of the amplified fragments were performed on ABI 3500xL Genetic Analyzer with data collection software, version 3.X.

### Single-cell DNA sequencing

PBMCs were sampled from two AML patients at pre-BMT, post-BMT and relapsed-AML time-points and kept in liquid N_2_ until use. Single-cell DNA sequencing libraries were generated from these PBMC samples with the Mission Bio Tapestri Single-cell DNA AML Kit according to manufacturer’s instruction (Mission Bio, South San Francisco, CA). Sequencing was performed with MiSeq Reagent 300-cycle V2 Kit (Illumina, San Diego, CA) by paired-end 2 × 150 cycles. Primary data processing was performed on DNANexus (www.dnanexus.com) while further bioinformatics analysis including data filtering and visualization was conducted using a R-based software package developed by Mission Bio. The targeted genes covered in the Mission Bio Tapestri Single-cell DNA AML Kit are indicated in Supplementary Table 2.

### Bulk next-generation DNA sequencing

Genomic DNA was extracted from the PBMC samples which were sampled from the same blood drawings as the ones for single-cell DNA sequencing with AllPrep DNA/RNA Mini kit according to manufacturer’s instruction (Qiagen Inc., Valencia, CA). Genomic DNA was stored at −20 °C until use.

Bulk sequencing libraries were generated with Illumina NGS Myeloid Panel Kit (Illumina, San Diego, CA) from the PBMC genomic DNA in the Stanford Clinical Molecular Genetic Pathology Laboratory. DNA sequencing and bioinformatics analysis were also conducted in the Stanford Clinical Molecular Genetic Pathology Laboratory. The targeted genes covered in Illumina NGS Myeloid Panel are shown in Supplementary Table 2. NextGENe (SoftGenetics, State College, PA) was used for data analysis.

### Single-cell sequencing data analysis

Data was analyzed using DNAnexus (www.dnanexus.com) and R software (www.r-project.org). The genotype caller used was freebayes (https://arxiv.org/abs/1207.3907) with optimized input parameters to output a VCF file. The VCF file was used together with Tapestri Analysis, a R-based application that facilitates data filtering and visualization. Cells and variants were filtered according to the following filter parameters: 1). Variant quality score (set at 30). 2). Read depth per cell per variant (minimum of 10 reads per variant in each cell). 3). Fraction of cells with missing/filtered data. 4). Fraction of variants with missing/filtered data. Cells with more than 7.5% missing data across all called variants were excluded from downstream analysis. Allele dropout rate was estimated with the help of data derived from allele-dropout (ADO) amplicons. In short, the fraction of cells that exhibited a non-heterozygous genotype call at a known heterozygous site represents the calculated ADO rate. The average signal across at least three loci was used for an average ADO computation.

For data visualization, R version 3.5.1 (www.r-project.org) was used. We used the heatmap2 function from the gplots package (ref^28^, http://cran.r-project.org) to present the data in heatmap format. The Rtsne package was utilized to visualize the data in t-SNE space (default parameters).

## Supplementary information

Supplementary Information
