# Supplementary material for: Clonal Evolution and Changes in Two AML Patients Detected with A Novel Single-Cell DNA Sequencing Platform
Source: Sci Rep. 2019 Jul 31;9:11119. doi: 10.1038/s41598-019-47297-z (PMC6668401; doi:10.1038/s41598-019-47297-z)

Supplementary Information

Clonal Evolution and Changes in Two AML Patients Detected with A Novel Single-Cell DNA Sequencing Platform

Liwen Xu[1], Robert Durruthy-Durruthy[2], Dennis J. Eastburn[2], Maurizio Pellegrino[2], Omid Shah[3], Everett Meyer[3], James Zehnder*[1]

[1]Department of Pathology, Stanford University, Stanford, CA 94305

[2]Mission Bio, Inc., South San Francisco, CA 94080

[3]Division of Blood and Marrow Transplantation. Department of Medicine, Stanford University, Stanford, CA 94305

Table of Contents:

Supplementary Table 1. Timeline of sample collection and patients' clinical information 3

Supplementary Table 2. Genes covered in Illumina NGS Myeloid Panel and Mission Bio Tapestri Single-cell DNA AML Panel 4

Supplementary Table 3: List of detected variants after quality control filtering 5

Supplementary Table 4: Changes of both AML status and oncogenic clone of cells 7

Supplementary Figure 1: Schematic of Mission Bio Tapestri single-cell NGS technology 8

Supplementary Figure 2: Quality control of single-cell sequencing data analysis 9

Supplementary Figure 3: *TP53* (c.379T>A) variant quality control (Patient 1) 10

Supplementary Table 1. Timeline of sample collection and patients' clinical information. The patients' clinical information was extracted from the routine clinical records at Stanford Health Care. Results at pre-BMT are highlighted in blue, at post-BMT in yellow, and at relapsed-AML in orange.

A. Timeline of sample collection and clinical information for Patient 1.

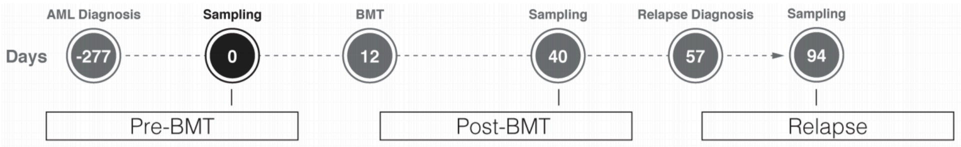

PBMC Collection Time	Pre-BMT	Post-BMT	Relapsed-AML
**Clinical Condition**	277 days after diagnosis & 12 days before BMT	28 days after BMT & 17 days before AML relapse	Relapse after 45 days of BMT. 37 days after diagnosis of relapse
**Treatments**	Hypomethalating agents	Cyclosporine & steroids	Hypomethalating agents

B. Timeline of sample collection and clinical information for Patient 2.

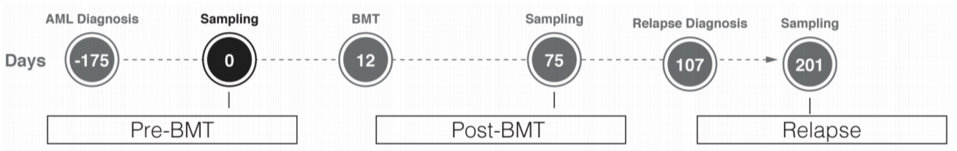

PBMC Collection Time	Pre-BMT	Post-BMT	Relapsed-AML
**Clinical Condition**	175 days after diagnosis & 12 days before BMT	63 days after BMT & 32 days before AML relapse	Relapse after 95 days of BMT. 95 days after diagnosis of relapse
**Treatments**	Cytotoxic chemo, hypomethalating agents, & lenalidomide	Cyclosporine & steroids	Hypomethalating agents

Supplementary Table 2. Genes covered in Illumina NGS Myeloid Panel and Mission Bio Tapestri Single-cell DNA AML Panel. Genes covered in both panels are in bold. Genes covered only in Illumina NGS Myeloid Panel but not in Mission Bio Tapestri Single-cell DNA AML Panel are not bolded.

**ASXL1**	**NPM1**	**DNMT3A**	**NRAS**
**EZH2**	**PTN11**	**FLT3**	**RUNX1**
**GATA2**	**SF3B1**	**IDH1**	**SRSF2**
**IDH2**	**TP53**	**JAK2**	**U2AF1**
**KIT**	**WT1**	**KRAS**	ABL1
ETV6/TEL	KDM6A	RAD21	ATRX
FBXW7	SETBP1	BCOR	BCORL1
MLL (KMT2A)	GATA1	MPL	SMC1A
BRAF	MYD88	SMC3	CALR
GNAS	NOTCH1	CBL	HRAS
STAG2	CBLC	PDGFRA	CDKN2A
IKZF1	PHF6	CSF3R	PTEN
CUX1	JAK3	ZRSP2	

Supplementary Table 3. List of detected variants after quality control filtering. Variants highlighted in grey are the variants with differential genotypes between patient and donor samples. Variants highlighted in yellow indicate the verified pathogenic variants. Variant highlighted in orange is the non-verified pathogenic variant. For each patient, sequencing data across all three timepoints (pre-BMT, post-BMT, relapse) were used to identify all variants that are listed in the table.

A. List of detected variants in Patient 1.

Chrom	Pos	Ref	Alt	Amplicon	ClinVar
11	32414333	G	T	WT1_1	rs2234593
13	28592546	T	C	FLT3_1	rs17086226
13	28602256	C	T	FLT3_3	rs61944200
13	28602292	T	C	FLT3_3	rs75580865
7	148504717	G	del	EZH2_1	rs760581114
7	148504852	GACTTA	dup	EZH2_1	rs560966145
7	148506363	T	G	EZH2_2	rs10268879
10	5554293	T	C	chr10_5554291	rs10904520
10	77210191	C	T	chr10_77210191	rs7072873
16	55770629	C	T	chr16_55770629	rs12925175
6	17076840	C	A	chr6_17076840	rs7754231
6	40116264	T	G	chr6_40116264	rs12194361
6	62094287	A	T	chr6_62094287	rs2879962
17	7578551	A	T	TP53_3	TP53_3
17	7578115	T	C	TP53_1	rs1625895
2	198266943	C	T	SF3B1_1	rs788019
20	31022959	T	C	ASXL1_2	rs6058694
4	55599436	T	C	KIT_1	rs1008658
18	9750662	T	C	chr18_9750662	rs8091737
14	56969005	C	T	chr14_56969005	rs12587027

B. List of detected variants in Patient 2

Chrom	Pos	Ref	Alt	Amplicon	ClinVar
10	5554293	T	C	chr10_5554291	rs10904520
10	77210191	C	T	chr10_77210191	rs7072873
11	32414333	G	T	WT1_1	rs2234593
12	112888139	C	G	PTPN11_2	rs397507503
13	28592546	T	C	FLT3_1	rs17086226

13	28602256	C	T	FLT3_3	rs61944200
13	28602292	T	C	FLT3_3	rs75580865
6	40116264	T	G	chr6_40116264	rs12194361
17	7577559	G	C	TP53_2	rs28934573
2	198266834	T	C	SF3B1_1	rs559063155
16	55770629	C	T	chr16_55770629	rs12925175
17	7577180	C	T	TP53_4	
17	7578115	T	C	TP53_1	rs1625895
18	9750662	T	C	chr18_9750662	rs8091737
2	198266943	C	T	SF3B1_1	rs788019
2	209113049	A	del	IDH1_1	rs774469589
4	55599436	GTGAT	GTGAC,GCGAC	KIT_1	rs1008658
6	17076840	C	A	chr6_17076840	rs7754231
7	148504717	G	del	EZH2_1	rs760581114
7	148504860	GACTTA	dup	EZH2_1	rs560966145

Supplementary Table 4. Changes of both AML status and oncogenic clone of cells. Results at pre-BMT are highlighted in blue, at post-BMT in yellow, and at relapsed-AML in orange.

A. Changes of both AML status and oncogenic clone in Patient 1.

Sample and Test Type	Assay Date	Blast %	Clinic Status	Single-cell Seq Sampling Date	TP53 Mutated Clone % in Single-cell Seq
**BM, Aspirate, Manual Differential**	8/7/2015	10%	Pre-BMT	8/23/2015	8.94%
**BM, Flow**	10/19/2015	8%	Post-BMT	10/2/2015	42.08%
**BM, Core Biopsy, Flow**	11/30/2015	21.6%	Relapsed-AML	11/25/2015	65.82%

B. Changes of both AML status and oncogenic clone in Patient 2.

Sample and Test Type	Assay Date	Blast %	Clinic Status	Single-cell Seq Sampling Date	TP53/SF3B1 Mutated Clone % in Single-cell Seq
**BM, Aspirate, Manual Differential**	11/11/2011	No Morphologic Evidence of Disease	Pre-BMT	11/27/2011	0%/0.34%
**BM, Aspirate**	3/13/2012	No Morphologic Evidence of Disease	Post-BMT	2/10/2012	0.19%/0.83%
**BM, Aspirate & Biopsy**	6/15/2012	20%	Relapsed-AML	6/15/2012	8.77%/5.86%

Supplementary Figure 1. Schematic of Mission Bio Tapestri single-cell NGS technology. A. High-level overview of the experimental steps in the workflow. B. The two-step microfluidic workflow. Individual cells are first encapsulated with lysis buffer containing protease and incubated to promote proteolysis. Protease activity is then thermally inactivated and the single-cell droplets containing the cell lysate are paired with droplets containing PCR reagents and barcoded beads. Targeted amplification of 40 AML-specific amplicons across 19 genes and barcode-tagging are performed in individual single-cell droplets. PCR amplicons are processed using conventional sequencing library preparation chemistry for high-throughput DNA sequencing and sequenced in MiSeq.

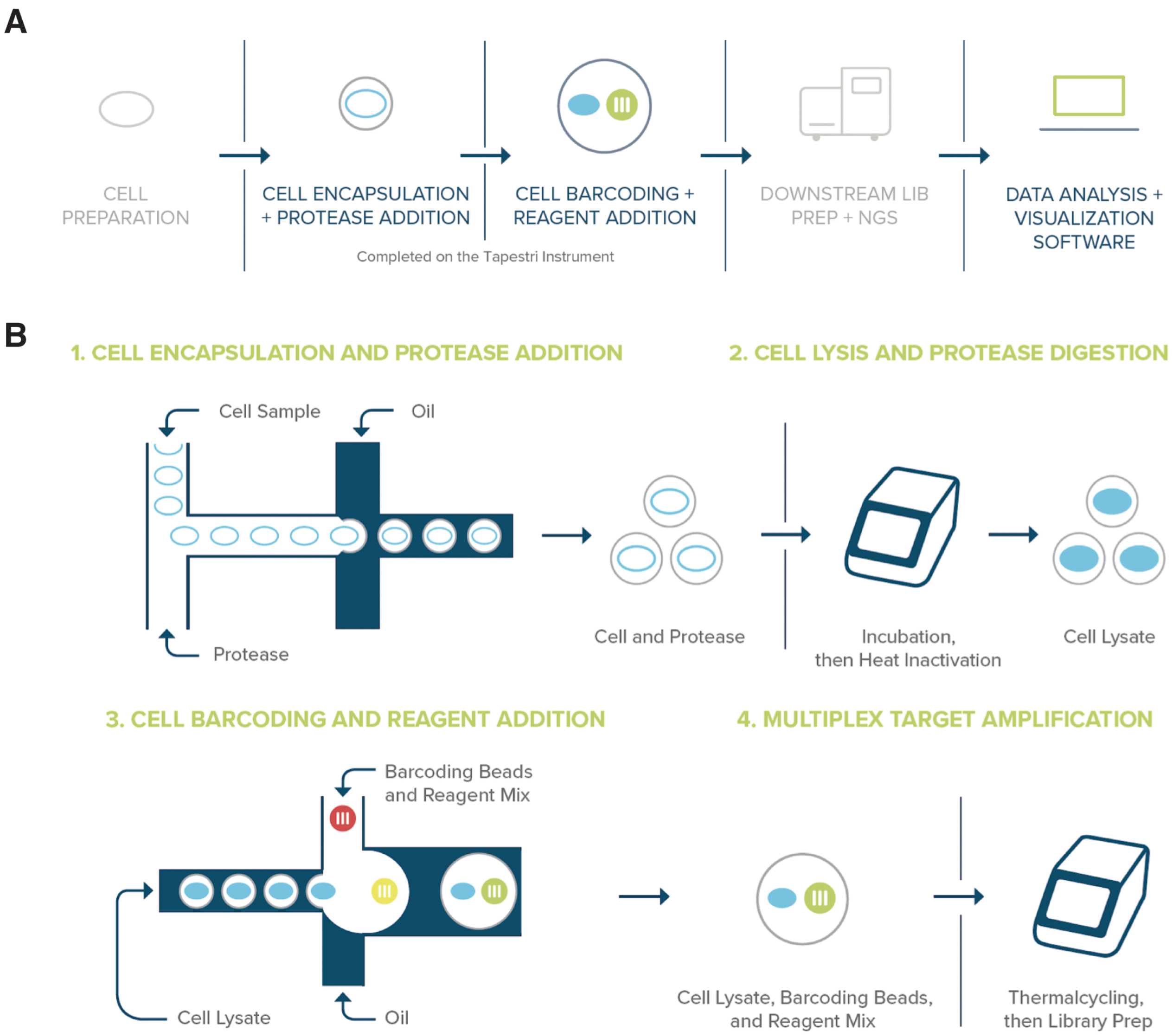

Supplementary Figure 2. Quality control of single-cell sequencing data analysis. A. Allele dropout (ADO) rates in each clinical sample. ADO rates were measured using two control amplicons (see Methods). **B. Percentage of missing data before and after quality control in each clinical sample. C. Histogram plots of cells with missing data.** For each time-point, the distribution of cells with missing data (N/A) is shown. Thresholds to exclude cells were set qualitatively based on the sample distribution and varied from 5 - 8% (in all cases cells with more than 8% of missing data were discarded from downstream analysis). The cells on the left side of the threshold lines were kept for downstream analysis and the cells on the right side of the threshold lines were excluded for downstream analysis. **D. Number and percentage of cells retained and discarded after quality control.** Number and percentage of cells in green are the cells that passed quality control and included for downstream data analysis. Number and percentage of cells in black are low quality cells (putative dead cells), which were filtered out and excluded from downstream analysis.

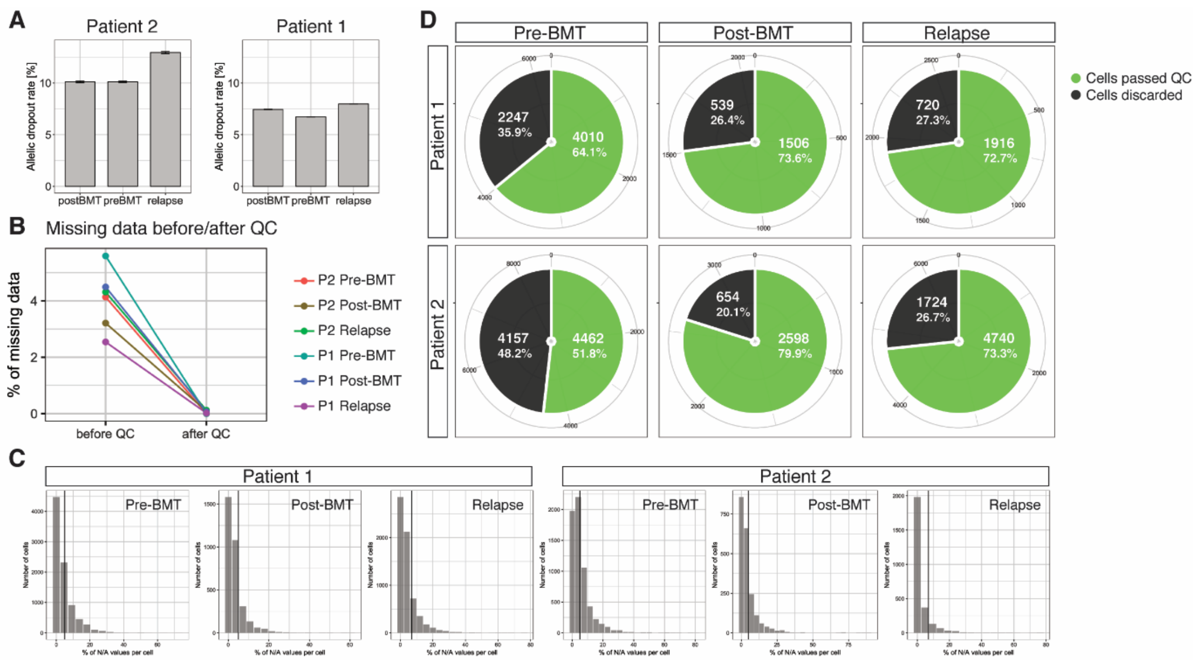

Supplementary Figure 3. *TP53* (c.379T>A) variant quality control (Patient 1). A. Sequencing read depth distributions per cell per variant. Data in each time-point are clustered (see Figure 1) and visualized in heatmap format. The number of reads per cell for each variant is color-coded ranging from white (min of 10 reads) to black (high number of reads). **B. Quantitative comparison of sequencing reads between *TP53* mutant cells and *TP53* wild-type cells.** Distributions shown in grey correspond to wild-type cells. Distributions shown in green correspond to *TP53* mutant cells. **C. Variant quality control comparison analysis.** Shown are distribution plots of variant quality score across all cells for each time-point. Dashed vertical lines correspond to the thresholds that were set to filter out low-quality variants (only variants with quality scores > 10^4 included). Vertical green lines correspond to quality scores of the *TP53* variant. Insets represent zoomed-in distribution plots showing variants with quality scores between 10^4 and 10^8. **D. Relationship between variant allele frequency and cell population frequency.**

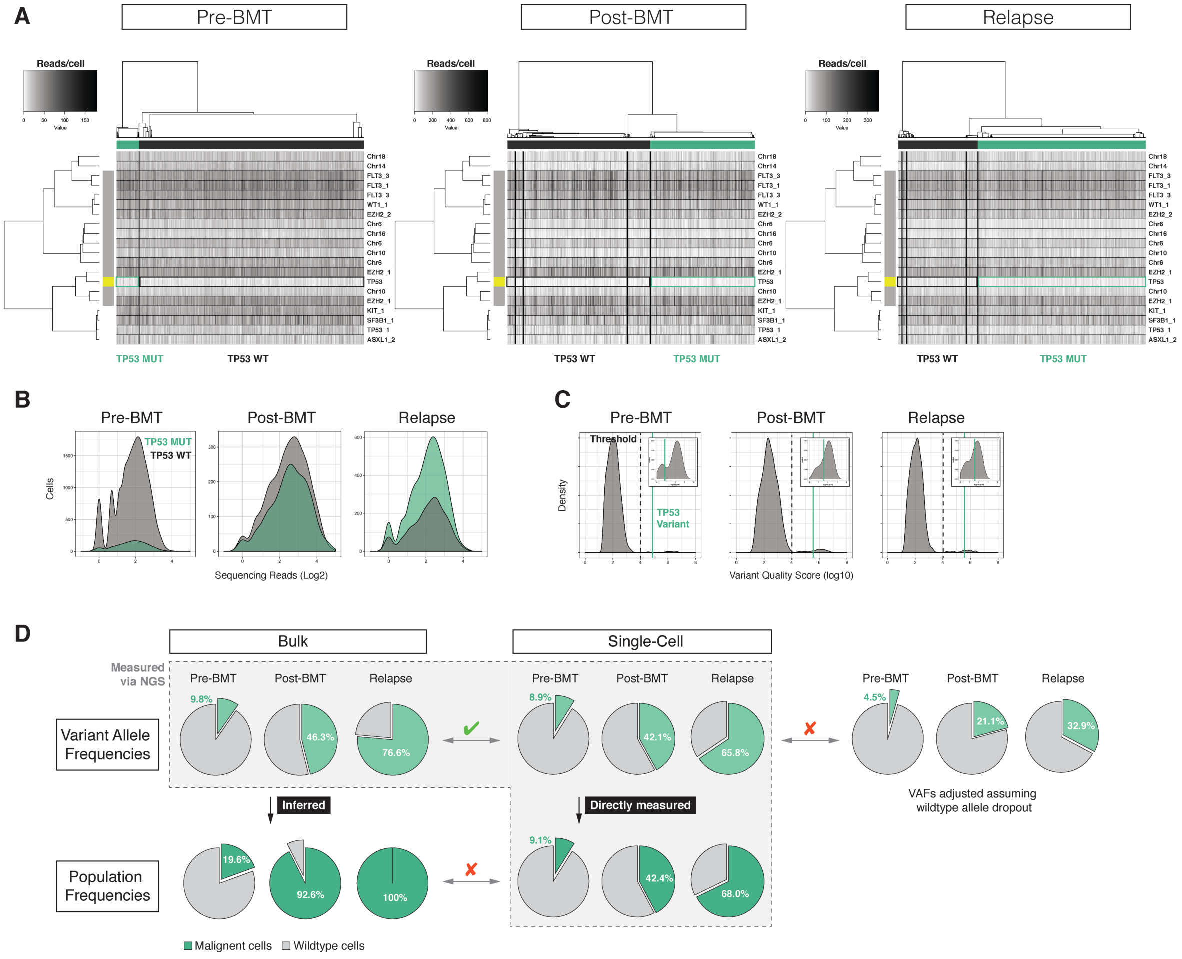

Supplement: Supplementary file 1 — Supplementary Information [file 41598_2019_47297_MOESM1_ESM.pdf]
